# Lactic acid bacteria-derived extracellular vesicles as postbiotic modulators of epithelial adhesion and barrier homeostasis: emerging perspectives

**DOI:** 10.3389/fmicb.2026.1905504

**Published:** 2026-07-29

**Authors:** Francesco Coppolino, Luca Tavella, Alessia Berbiglia, Francesco Simone Sframeli, Daniele Filippone, Federica Grasso, Agata Famà, Roberta Lucia Pecora, Maria Santagati, Germana Lentini, Giuseppe Valerio De Gaetano, Concetta Beninati

**Affiliations:** 1 Department of Human Pathology in Adult and Developmental Age "Gaetano Barresi", University of Messina, Messina, Italy; 2 Section Microbiology, Department of Biomedical and Biotechnological Sciences (BIOMETEC), University of Catania, Catania, Italy; 3 Scylla Biotech Srl, Messina, Italy

**Keywords:** epithelial adhesion, epithelial barrier, extracellular vesicles, host–microbe interaction, integrins, lactic acid bacteria, postbiotics, tight junctions

## Abstract

Microbiota-targeted strategies are increasingly moving beyond the administration of live microorganisms toward cell-free microbial components with postbiotic potential. Among these, extracellular vesicles released by lactic acid bacteria (LAB-derived EVs) are emerging as biologically active particles capable of transferring bacterial signals to host epithelial and immune cells. However, their effects should not be interpreted as uniform or intrinsically beneficial, because vesicle composition and activity are strongly influenced by bacterial strain, culture conditions, growth phase, isolation procedures and target cell context. In this Perspective, we discuss LAB-derived EVs as candidate postbiotic modulators of epithelial adhesion and barrier homeostasis. We focus on the epithelial interface as an integrated functional unit in which tight junctions, adherens junctions, junctional adhesion molecules, integrin-related cell–matrix adhesion, extracellular matrix remodeling and epithelial–immune communication converge. Evidence, largely derived from *Lactobacillus*/*Lactobacillaceae* models, supports the ability of LAB-derived EVs to influence barrier integrity, inflammatory tone, pathogen adhesion and tissue repair. We also propose an exploratory framework based on early transcriptional responses observed in A549 respiratory epithelial cells exposed to an EV-enriched preparation obtained from *Lactobacillus fermentum* 18A-TV, highlighting a transcriptional signature compatible with transient epithelial remodeling involving junctional, extracellular matrix, integrin-related and repair-associated genes. Future studies should combine standardized EV characterization with protein-level and functional assays to define reproducible potency markers and clarify the therapeutic potential of LAB-derived EVs.

## 1 Introduction

Microbiota-targeted strategies have traditionally relied on the administration of live microorganisms, particularly probiotics, to support mucosal health. However, the use of viable bacteria can be limited by strain viability, storage stability, manufacturing reproducibility and safety concerns in vulnerable individuals. These limitations have increased interest in cell-free microbial derivatives within the field of postbiotics. According to the ISAPP consensus definition, a postbiotic is a preparation of inanimate microorganisms and/or their components that confers a health benefit on the host (Salminen et al., 2021). This concept recognizes that microbial benefits may be mediated not only by viable cells, but also by structural components, metabolites, secreted factors and complex particles released by the parental organism. Among these candidate effectors, bacterial extracellular vesicles (EVs) have attracted increasing attention. EVs are nanosized lipid-bilayer particles released by both Gram-negative and Gram-positive bacteria and carrying a heterogeneous cargo that may include proteins, lipids, peptidoglycan fragments, nucleic acids, metabolites and microbial-associated molecular patterns. Through this cargo, bacterial EVs can participate in interbacterial communication and host–microbe interactions, acting through receptor engagement or after internalization by recipient host cells. Microbial EVs are therefore increasingly recognized as mediators of host–microbiota communication, influencing epithelial, immune and metabolic signaling pathways (Abubaker et al., 2024). Within this field, EVs derived from lactic acid bacteria (LAB) represent a relevant but still incompletely defined category of cell-free microbial effectors. LAB include *Lactobacillus sensu lato*, *Lactococcus* and related genera with probiotic, commensal or technological relevance (Zheng et al., 2020). Throughout this Perspective, the term LAB-derived EVs is used as an umbrella term for vesicles released by lactic acid bacteria and related *Lactobacillales* of probiotic, commensal or technological relevance. However, we acknowledge that the functional evidence currently available at epithelial interfaces is dominated by *Lactobacillus*/*Lactobacillaceae*-derived EVs, whereas direct studies on EVs from other LAB genera, including *Lactococcus*, *Pediococcus*, *Leuconostoc*, *Weissella* and selected *Enterococcus* species, remain limited. Therefore, conclusions regarding epithelial interface modulation should be considered primarily supported by *Lactobacillus*-associated evidence and should not be extrapolated uncritically to all LAB genera. It is vital to emphasize that EV biological activities are intrinsically linked to the unique molecular cargo of the parental organism; consequently, substantial phenotypic variations may occur not only between different genera or species, but even among distinct strains of the same species. Recent reviews have highlighted their role in intercellular communication and host interaction, together with unresolved issues related to isolation, cargo characterization and functional standardization (Li et al., 2024). In *Lactococcus lactis*, EV formation can be stimulated by a prophage-encoded holin–lysin system, supporting the idea that vesicle release in LAB may be strain- and condition dependent (Liu et al., 2022). A major biological interface for LAB-derived EV activity is the epithelium, where tight junctions, adherens junctions, junctional adhesion molecules, integrin-related cell–matrix adhesion and immune signaling maintain tissue homeostasis. Studies on probiotic or commensal bacterial EVs, particularly in intestinal models, indicate effects on tight junction-associated proteins, permeability and inflammatory signaling (Li et al., 2024; Krzyżek et al., 2023; González-Lozano et al., 2022). Nevertheless, the current literature does not support a uniform effect of LAB-derived EVs across tissues and models. Their activity appears context-dependent, varying according to bacterial species or strain, vesicle preparation, epithelial compartment and inflammatory or homeostatic state. The conceptual aim of this Perspective is to organize this heterogeneous evidence within an epithelial interface homeostasis framework. Previous reviews have mainly discussed LAB-derived or probiotic-derived EVs in relation to general postbiotic activity, barrier protection, immunomodulation or host–microbe interactions. Here, we propose a more integrated view in which these processes are considered as interconnected components of the same epithelial interface. In this framework, tight junctions, adherens junctions, junctional adhesion molecules, cell–matrix anchoring, extracellular matrix remodeling, epithelial immune sensing and repair-associated responses are not treated as isolated endpoints, but as functionally linked modules that together define epithelial homeostasis. Accordingly, the novelty of this Perspective does not lie in proposing a single conserved mechanism of LAB-derived EV action across all tissues or strains. Rather, it lies in reframing available evidence around a shared biological question: how LAB-derived EVs may influence the epithelial interface as an integrated functional unit. This framework is intended to clarify the scope of current evidence, identify underexplored components such as integrin-related cell–matrix adhesion and extracellular matrix remodeling, and define testable directions for future studies.

## 2 LAB-derived EVs as candidate postbiotic particles: biological complexity and methodological constraints

LAB-derived extracellular vesicles should be interpreted as biologically structured but heterogeneous particles rather than as uniform postbiotic entities. Although the study of bacterial vesicles was initially dominated by Gram-negative outer membrane vesicles, it is now well established that Gram-positive bacteria, including lactic acid bacteria, can also release extracellular vesicles despite the presence of a thick peptidoglycan cell wall. In these organisms, vesicle release may involve local cell wall remodeling, peptidoglycan turnover, autolysin- or endolysin-associated processes and adaptive responses to metabolic or envelope stress (Li et al., 2024; Brown et al., 2015; Briaud and Carroll, 2020; Xu et al., 2023). In *L. lactis*, extracellular vesicle formation has also been linked to a prophage-encoded holin–lysin system, supporting the idea that EV release in LAB can be strain- and condition-dependent rather than governed by a single conserved pathway (Liu et al., 2022). The potential postbiotic relevance of LAB-derived EVs is closely related to their cargo. These particles may contain membrane-associated proteins, enzymes, lipids, nucleic acids, metabolites and cell-envelope-derived components. Proteomic studies on *Lactobacillus* and related LAB-derived EVs have identified proteins associated with catalytic activity, carbohydrate metabolism, cell wall organization, adhesion and stress responses, suggesting that these vesicles are not undefined cellular debris but selectively enriched microbial particles (Li et al., 2024; Krzyżek et al., 2023; Lee et al., 2023). Available characterization studies indicate that LAB-derived EVs may contain a heterogeneous but functionally relevant molecular cargo. Reported protein components include membrane-associated and cell wall-associated proteins, surface-layer or adhesion-related proteins, autolysins, stress-response proteins, metabolic enzymes and so-called moonlighting proteins such as glyceraldehyde-3-phosphate dehydrogenase and enolase. These proteins may contribute to vesicle stability, epithelial interaction, immune recognition or other host–microbe communication processes, although their functional contribution must be demonstrated experimentally for each preparation (Li et al., 2024). In addition to proteins, LAB-derived EV preparations may contain lipid and cell-envelope-associated components, including membrane phospholipids, glycolipids, peptidoglycan-derived fragments and lipoteichoic acid or related microbial-associated molecular patterns (Li et al., 2024). Nucleic acid cargo has also been reported in bacterial EV preparations, including small RNA species and DNA fragments, but its abundance, stability and biological relevance remain preparation-dependent and require careful validation. Other bioactive components, such as metabolites, organic acids or antimicrobial peptide-like molecules, may also be associated with specific preparations (Li et al., 2024; Dean et al., 2019). Overall, LAB-EV composition should be considered strain-, growth condition- and isolation-dependent, and no single cargo profile can currently be generalized to all LAB-derived EVs.

A selected overview of reported physicochemical features, molecular cargo and biological activities of LAB-derived EV preparations is provided in Table 1. This table is intended to summarize the heterogeneity of available evidence rather than to imply that a common cargo profile applies to all LAB-derived EVs.

**Table 1:** Selected physicochemical features, molecular cargo and biological activities reported for LAB-derived extracellular vesicle preparations.

	
*Lactobacillus fermentum* 18A-TV EV-enriched fraction	
Reported particle features and isolation approach	EV-enriched fraction obtained from 60 h MRS culture by centrifugation, 0.22 μm filtration and sequential ultracentrifugation; predominant nanoscale population around 46.8 nm by number distribution; derived count rate 797.4; protein content 1,424 ng/μL; DNA content 0.804 ng/μL.
Protein cargo	Total protein content measured; specific protein cargo not yet characterized.
Nucleic acid cargo	Low DNA content detected; RNA cargo not yet characterized.
Lipid/cell-envelope-associated cargo	Lipid and cell-envelope-associated cargo not yet characterized.
Bioactive molecules/candidate effectors	EV-enriched fraction selected because it showed the strongest vesicle-associated signal among tested culture time points.
Reported biological functions	Early, transient A549 transcriptional response involving junctional, extracellular matrix, integrin-related and remodeling-associated genes; requires protein-level and functional validation.
Reference	Present study; Supplementary methods.
*Lactobacillus casei* BL23 EVs	
Reported particle features and isolation approach	EVs isolated from 48 h MRS culture supernatant by filtration and ultracentrifugation; TEM showed spherical-like EVs with narrow size distribution, 13–28 nm, mean diameter 19.21 ± 2.51 nm; DLS showed a main small-vesicle population around 32.84 ± 4.4 nm by intensity and mode 24.36 nm by number; PEG6000-isolated EVs showed slightly larger particles, around 50.75 nm by intensity and 43.82 nm by number, with possible aggregates requiring filtration.
Protein cargo	P40 and P75 detected by Western blot and localized on the EV surface by proteinase K assay; estimated contribution to total EV protein load: P40 1.9 ± 0.9%, P75 7.4 ± 1.2%; recombinant fluorescent proteins could also be detected in EVs from engineered BL23 strain.
Nucleic acid cargo	Not specifically characterized for natural RNA/DNA cargo in the cited study.
Lipid/cell-envelope-associated cargo	Lipoteichoic acid detected and quantified in BL23 EVs; mean 58.913 ± 3.393 ng LTA/μg EV protein.
Bioactive molecules/candidate effectors	Surface-exposed P40 and P75; LTA; EV-associated proteins capable of engaging epithelial signaling.
Reported biological functions	Stimulation of EGFR pathway and downstream p-Akt/p-ERK signaling in intestinal epithelial cells; no clear anti-inflammatory activity in the HT29 SEAP assay after TNF-α or IL-1β stimulation.
Reference	Bäuerl et al. (2020)
*Lactobacillus kefirgranum* PRCC-1301 EVs	
Reported particle features and isolation approach	EVs obtained from culture supernatant after growth in glucose base medium for 17–24 h to OD600 1.0; bacterial cells removed by centrifugation and filtration; EVs isolated/concentrated by ultrafiltration; particle size not reported in the available methods excerpt.
Protein cargo	Specific protein cargo not extensively characterized in the available text.
Nucleic acid cargo	Nucleic acid cargo not reported.
Lipid/cell-envelope-associated cargo	Lipid or cell-envelope-associated cargo not reported.
Bioactive molecules/candidate effectors	EV-enriched/ultrafiltered preparation derived from a kefir-associated *Lactobacillus kefirgranum* strain.
Reported biological functions	Attenuation of intestinal inflammatory responses and preservation/restoration of tight junction-associated markers, including ZO-1, occludin and claudin-1, in DSS-related intestinal models.
Reference	Kang et al. (2020)
*Lactobacillus crispatus* BC5/*Lactobacillus gasseri* BC12 EVs	
Reported particle features and isolation approach	EVs isolated from stationary-phase anaerobic MRS cultures by centrifugation, 0.22 μm filtration, debris removal and ultracentrifugation; characterized by NTA and DLS. *Lactobacillus crispatus* BC5-EVs: 34.70 ± 2.31 × 10^^9^ particles/mL, mean size 89.3 ± 49.2 nm, ζ-potential −20.3 ± 1.8 mV. *Lactobacillus gasseri* BC12-EVs: 30.60 ± 1.05 × 10^9 particles/mL, mean size 129.1 ± 51.9 nm, *ζ*-potential −10.4 ± 0.7 mV. Sterile MRS-derived particles were also characterized as medium control.
Protein cargo	Total protein content measured and normalized per 10^9 particles: 1.19 ± 0.07 μg/10^^9^ particles for BC5-EVs and 1.83 ± 0.27 μg/10^^9^ particles for BC12-EVs; specific protein cargo not further resolved.
Nucleic acid cargo	DNA and RNA detected and quantified: BC5-EVs contained 0.22 ± 0.04 μg DNA and 0.03 ± 0.01 μg RNA per 10^9 particles; BC12-EVs contained 0.11 ± 0.00 μg DNA and 0.08 ± 0.03 μg RNA per 10^^9^ particles.
Lipid/cell-envelope-associated cargo	Lipid composition and specific cell-envelope-associated cargo not extensively characterized.
Bioactive molecules/candidate effectors	EV preparations containing proteins, DNA and RNA; no specific molecular effector identified; inclusion of MRS-derived particles highlights the relevance of medium-only controls.
Reported biological functions	Enhanced adhesion of beneficial lactobacilli to epithelial cells and reduced adhesion of opportunistic pathogens, including *Escherichia coli*, *Staphylococcus aureus*, *Streptococcus agalactiae* and *Enterococcus faecalis*, in cervicovaginal epithelial/HeLa adhesion models.
Reference	Croatti et al. (2022)
*Lactobacillus plantarum* APsulloc 331261 EVs	
Reported particle features and isolation approach	EVs purified from anaerobic MRS cultures by sequential centrifugation, 0.45 μm filtration, tangential flow filtration and ultracentrifugation; further purified by OptiPrep density gradient, with EV-enriched fractions collected at densities of 1.17–1.24 g/mL. TRPS analysis showed a diameter of approximately 104 ± 42.4 nm, mode 77 nm, for UC-purified LEVs and 83 ± 20.3 nm, mode 73 nm, for density-purified LEVs. Mean particle number per mg protein was 2.13 × 10^9 for UC-purified LEVs and 3.83 × 10^10 for density-purified LEVs. Bio-TEM and cryo-EM showed a closed spherical membrane structure.
Protein cargo	Total protein concentration measured by Bradford assay; specific protein cargo not further characterized in the cited study.
Nucleic acid cargo	Nucleic acid cargo not reported.
Lipid/cell-envelope-associated cargo	Lipid or cell-envelope-associated cargo not reported.
Bioactive molecules/candidate effectors	LEVs purified according to a MISEV2018-oriented workflow; unconditioned MRS medium concentrates processed in parallel as negative controls.
Reported biological functions	Anti-inflammatory and immunomodulatory activity in human skin organ cultures and THP-1 cells; suppression of multiple inflammatory cytokines, increased IL-10 secretion and induction of monocyte-to-macrophage differentiation with increased CD14 and ICAM-1 and reduced CCR2 expression.
Reference	Kim et al. (2020)
*Lactobacillus rhamnosus* GG EVs	
Reported particle features and isolation approach	EVs isolated from LGG ATCC 53103 culture supernatants after cultivation in MRS medium through sequential culture steps of 12 h, 8 h and 24 h, followed by centrifugation, 0.22 μm filtration, ultracentrifugation at 150,000 × *g*, ultrafiltration through a 50–100 kDa membrane and a second ultracentrifugation step. LGG-EVs showed sphere-shaped morphology by TEM; NTA showed a particle size range of 30–200 nm, with a main peak around 180 nm; zeta potential was approximately −19.4 mV.
Protein cargo	Total EV protein concentration measured by BCA assay; specific protein cargo not extensively characterized in the cited study.
Nucleic acid cargo	miRNA cargo characterized by high-throughput small RNA sequencing; 494 miRNAs were identified, including 193 known and 301 novel miRNAs. miR-21-5p was among the highly expressed repair-associated miRNAs and was functionally validated by transfer to HUVEC and HaCaT cells and by miR-21-5p inhibitor experiments.
Lipid/cell-envelope-associated cargo	Lipid or cell-envelope-associated cargo not reported.
Bioactive molecules/candidate effectors	EV-associated miRNA cargo, particularly miR-21-5p, implicated in repair-associated signaling; LGG-EVs activated AKT/HIF1α signaling in endothelial cells and keratinocytes, at least in part through miR-21-5p transfer.
Reported biological functions	Promotion of cutaneous wound healing in a full-thickness mouse wound model; enhanced re-epithelialization, granulation tissue formation, collagen deposition, wound cell proliferation and angiogenesis, with increased Ki67, CD31, Vegfa and Hif1a signals; LGG-EVs also enhanced proliferation, migration and tube formation in HUVEC and proliferation/migration in HaCaT cells.
Reference	Wang et al. (2024)
*Lactococcus lactis-*derived EVs	
Reported particle features and isolation approach	EVs produced by *Lactococcus lactis* strains FM-YL11, prophage-cured FM-YL12 and holin–lysin mutant FM-YL11ΔHLH under prophage-inducing or non-inducing conditions; prophage induction performed with mitomycin C. EVs were isolated from culture supernatants by low-speed centrifugation, pH adjustment, 0.45 μm filtration and ultracentrifugation at 160,000 × *g*. TEM showed EV-like structures ranging from approximately 50–300 nm; EV-like structures from prophage-cured FM-YL12 were generally smaller than 150 nm. Phage head-like particles of approximately 50 nm were also observed under some conditions.
Protein cargo	EV protein content measured by BCA; EV proteome analyzed by LC–MS/MS and label-free iBAQ-based proteomics. Prophage-encoded holin and lysin genes were mechanistically implicated in EV formation, but should not be interpreted as EV cargo unless specifically detected in EV fractions.
Nucleic acid cargo	DNA-associated signals assessed by DAPI staining after DNase treatment. Distinct DNA-positive populations were observed in prophage-induced FM-YL11 EV fractions, including a high-DNA-signal population likely corresponding to phage head-like particles or tailless phage-related structures; therefore, DNA signals should be interpreted cautiously.
Lipid/cell-envelope-associated cargo	Membrane-associated particles detected using FM4-64 membrane dye; lipid composition not specifically characterized.
Bioactive molecules/candidate effectors	Prophage-encoded holin–lysin system identified as a driver of EV formation; mitomycin C-induced prophage activation increased EV production approximately 10-fold in FM-YL11, whereas prophage-cured and holin–lysin mutant strains did not show the same increase; holin–lysin complementation restored enhanced EV production.
Reported biological functions	EV biogenesis study showing that vesicle formation in *Lactococcus lactis* can be prophage-, holin–lysin-, strain- and condition-dependent; not primarily an epithelial functional or postbiotic activity study.
Reference	Liu et al. (2022)

DLS, dynamic light scattering; EVs, extracellular vesicles; LGG, *Lactobacillus rhamnosus* GG; LEVs, *Lactobacillus plantarum*-derived extracellular vesicles; LTA, lipoteichoic acid; NTA, nanoparticle tracking analysis; TEM, transmission electron microscopy; TRPS, tunable resistive pulse sensing; UC, ultracentrifugation.

NR/not reported indicates that the corresponding cargo class was not specifically analyzed, not reported in sufficient detail or not central to the cited study.

The inclusion of sterile MRS-derived particles in some studies highlights the importance of medium-only controls, because culture media may contribute particle-associated proteins, nucleic acids or other co-isolated material that can confound the interpretation of bacterial EV preparations.

For *Lactococcus lactis*, the cited study is included as an example of LAB EV biogenesis rather than epithelial functional activity. Because prophage induction generated EV-like structures together with phage head-like or phage-related particles, cargo-associated DNA signals should be interpreted in the context of prophage biology and not as generic evidence of nucleic acid cargo in LAB-derived EVs.

Overall, Table 1 highlights that the molecular characterization of LAB-derived EVs remains uneven across studies. Protein cargo is the most frequently investigated component, whereas nucleic acids, lipid composition, cell-envelope-derived structures and soluble co-isolates are less consistently analyzed. This heterogeneity reinforces the need for standardized characterization before assigning specific epithelial functions to defined EV cargo components. At the same time, their composition is influenced by bacterial strain, growth phase, culture conditions, vesicle biogenesis pathway and isolation procedure, all of which may affect biological activity, reproducibility and safety (Li et al., 2024; Krzyżek et al., 2023; Park et al., 2025).

Unlike Gram-negative outer membrane vesicles, LAB-derived vesicles do not contain lipopolysaccharide, reflecting the absence of an outer membrane in Gram-positive bacteria. This feature may represent an advantage for postbiotic development, but it does not eliminate the need for careful safety and functional assessment. Co-isolated soluble microbial products, medium-derived particles or protein aggregates may contribute to biological responses if purification and control strategies are not rigorous. Therefore, standardized isolation, orthogonal physicochemical characterization and appropriate EV-depleted and medium-only controls are essential before assigning a specific function to LAB-derived EV preparations (Lötvall et al., 2014). The interpretation of LAB-derived EV studies should also be considered in light of evolving international EV standardization frameworks. MISEV2014 provided initial minimal information guidelines for EV studies, whereas MISEV2018 expanded recommendations on EV nomenclature, source description, separation methods, characterization, quantification, contaminant assessment and functional studies (Théry et al., 2018). More recently, MISEV2023 further updated these recommendations by incorporating advances in EV production, separation, characterization, release, uptake and functional analysis, while emphasizing transparency, reproducibility and the reporting of method-specific limitations (Welsh et al., 2024).

Although MISEV guidelines were mainly developed for the broader EV field, their principles are highly relevant to bacterial and LAB-derived EV research. In this setting, specific challenges include the possible co-isolation of soluble bacterial products, protein aggregates, medium-derived particles, cell wall fragments, nucleic acids or other non-vesicular components. These contaminants may contribute to biological responses and should be carefully controlled, particularly when EVs are proposed as candidate postbiotic effectors.

Future studies should therefore combine complementary separation and analytical approaches. Size-exclusion chromatography and density-gradient purification may reduce co-isolation of soluble proteins and medium-derived contaminants compared with crude or single-step enrichment procedures. Asymmetric flow field-flow fractionation may improve the resolution of heterogeneous nanoparticle populations, whereas nano-flow cytometry and other single-vesicle approaches can support particle counting and phenotyping at higher resolution (Welsh et al., 2024). Cryo-electron microscopy can provide structural information on vesicle morphology, while single-vesicle fluorescence analysis may help distinguish vesicle-associated signals from bulk contaminants (Welsh et al., 2024). Together with EV-depleted controls, medium-only controls, dose–response designs and functional assays, these approaches will be important for defining reproducible potency markers and for distinguishing vesicle-associated effects from responses induced by non-vesicular bacterial products (Welsh et al., 2024). Their value as candidate postbiotic mediators does not depend on assuming a predefined mechanism of action, but on demonstrating reproducible preparation, defined cargo features and measurable functional effects at the epithelial interface.

## 3 LAB-derived EVs at epithelial interfaces: barrier regulation, adhesion and immune dialogue

The epithelial interface represents a major biological target for LAB-derived EV activity. Epithelial surfaces are not passive physical barriers, but dynamic tissues in which permeability control, microbial sensing, immune communication, cell–cell cohesion and cell–matrix anchoring are continuously integrated. For this reason, the concept of epithelial adhesion should be interpreted broadly. It does not refer only to the attachment of microorganisms to epithelial cells, but also to the molecular systems that maintain epithelial integrity and connect epithelial cells to the extracellular matrix. Cell–cell adhesion is maintained by tight junctions, adherens junctions and junctional adhesion molecules (Buckley and Turner, 2018). Tight junctions regulate paracellular permeability and epithelial polarity through the coordinated activity of transmembrane proteins such as claudins, occludin and junctional adhesion molecules, together with cytoplasmic scaffold proteins such as zonula occludens proteins (France and Turner, 2017). In this network, OCLN contributes to tight junction organization and barrier regulation, whereas F11R/JAM-A participates in junctional assembly, epithelial cohesion and permeability control. Adherens junction components, including E-cadherin and catenins, further contribute to epithelial cohesion and cytoskeletal organization. In parallel, cell–matrix adhesion is largely mediated by integrins, which connect extracellular matrix components to intracellular cytoskeletal and signaling networks (Anderson et al., 2014). Integrin β1-containing complexes are among the major epithelial adhesion systems, whereas α_2_β_1_ integrin is classically involved in interactions with collagen-rich matrices (De Gaetano et al., 2022, 2024). Thus, modulation of junctional molecules, extracellular matrix components and integrin-related pathways may indicate changes in how epithelial cells maintain barrier architecture, sense their microenvironment and activate repair-associated responses. This integrated view is particularly relevant for LAB-derived EVs because their molecular cargo can expose epithelial cells to complex microbial signals in a protected and spatially organized form. By carrying membrane-associated proteins, lipids, nucleic acids, cell-envelope-derived structures, metabolites and other microbial-associated molecular patterns, EVs may influence epithelial physiology through surface receptor engagement, intracellular uptake and downstream activation of barrier-, adhesion- and immunity-associated pathways (Abubaker et al., 2024; Li et al., 2024; Krzyżek et al., 2023). Available evidence links LAB-derived EVs to selected molecular components of epithelial barrier regulation, inflammatory tuning and epithelial–immune communication, although the strength of evidence differs substantially across targets and models. In intestinal inflammatory models, EVs from kefir-associated *Lactobacillus kefirgranum* PRCC-1301 have been associated with attenuation of inflammatory signaling and preservation or restoration of tight junction-related proteins, including ZO-1, occludin and claudin-1, in connection with modulation of NF-κB-dependent responses (Kang et al., 2020). Other *Lactobacillus*-derived EV studies have reported attenuation of inflammatory mediators, including TNF-α and IL-6, and modulation of anti-inflammatory or regulatory responses such as IL-10-associated pathways, depending on the experimental model (Kim et al., 2018, 2020; Choi et al., 2020; Tong et al., 2021; Hao et al., 2021).

Beyond classical tight-junction markers, available evidence also suggests that LAB-derived EVs may influence epithelial repair, surface conditioning and epithelial–immune interaction. For example, *Lactobacillus casei* BL23-derived EVs were reported to stimulate EGFR-related epithelial survival and repair-associated signaling, likely through vesicle-associated P40 and P75 proteins (Bäuerl et al., 2020). In vaginal epithelial models, EVs from *Lactobacillus crispatus* BC5 and *Lactobacillus gasseri* BC12 enhanced beneficial lactobacilli adhesion and reduced pathogen attachment, supporting the concept that LAB-derived EVs may condition epithelial surfaces (Croatti et al., 2022). In skin-related and immune-cell models, *Lactobacillus plantarum*-derived EVs have been reported to reduce inflammatory damage and IL-6 production in epithelial and immune cell systems and to promote anti-inflammatory macrophage polarization, including M2-like responses (Kim et al., 2018, 2020; Gong et al., 2024). In parallel, *Lactobacillus rhamnosus* GG-derived EVs have been associated with wound-healing responses involving re-epithelialization and angiogenesis (Wang et al., 2024). At the same time, direct evidence is not equally available for all components of the epithelial interface. Molecules such as JAM-A/F11R, E-cadherin, β-catenin, ICAM-1, VCAM-1, NOD2 and integrin-related adhesion pathways are biologically relevant to epithelial interface homeostasis, but their direct regulation by LAB-derived EVs remains incompletely characterized or model-dependent. We therefore include these molecules within the conceptual framework as important nodes connecting barrier integrity, adhesion, immune dialogue and repair, while recognizing that their modulation requires targeted validation in future studies. Pattern-recognition pathways, including TLR2-, TLR4- or NOD-related signaling, may also contribute to epithelial and immune responses to bacterial EV cargo, but their specific involvement in LAB-derived EV activity requires preparation- and model-specific validation.

Overall, the available evidence identifies three recurrent, although not universal, functional domains of LAB-derived EV activity at epithelial interfaces. First, intestinal studies support a role for selected *Lactobacillus*-derived EVs in barrier and inflammatory tuning, including modulation of tight junction-associated proteins, inflammatory signaling and cytokine responses. Second, vaginal models indicate that EVs released by symbiotic lactobacilli may condition epithelial surfaces, favor beneficial bacterial adhesion and interfere with pathogen attachment or viral entry (Croatti et al., 2022; Ñahui Palomino et al., 2019). Third, skin-related models suggest that LAB-derived EVs may contribute to epithelial protection, immune modulation and repair-associated responses, including macrophage polarization, re-epithelialization and angiogenesis. These domains provide the functional basis for the epithelial interface homeostasis framework, but they should be interpreted as strain-, preparation- and model-dependent effects rather than as a conserved mechanism shared by all LAB-derived EVs. Regarding integrins, it is important to distinguish between their relevance within the epithelial interface framework and the current level of direct evidence available for LAB-derived EVs. Integrins are central mediators of epithelial cell–matrix adhesion, extracellular matrix sensing and repair-associated signaling (Anderson et al., 2014); therefore, they represent biologically relevant components of epithelial interface homeostasis. However, direct evidence demonstrating that LAB-derived EVs regulate integrin expression, localization or signaling remains limited. For this reason, integrin-related pathways are not presented here as an established mechanism of LAB-derived EV action. Rather, they are proposed as an important future research direction emerging from the broader epithelial interface framework and from the exploratory A549 transcriptional observations. Future studies should determine whether LAB-derived EVs can modulate integrin protein expression, integrin localization, cell–matrix adhesion, focal adhesion signaling or epithelial repair responses through targeted functional assays. A concise overview of selected studies supporting the role of LAB-derived EVs in epithelial barrier regulation, adhesion, immune modulation and repair is provided in Table 2. Rather than implying that all LAB-derived EVs exert the same biological effect, the studies summarized in the table highlight recurring functional domains that provide a conceptual basis for the prospective respiratory epithelial framework discussed below. Each study is linked to the corresponding numbered reference to facilitate direct comparison with the cited literature.

**Table 2:** Selected evidence supporting LAB-derived EVs as context-dependent modulators of epithelial interface homeostasis.

	
EV biogenesis/methodological context	
EV source	*Lactococcus lactis*
Model	Bacterial model/EV biogenesis
Epithelial interface	EV production
Main reported effect	EV formation stimulated by prophage-associated mechanisms
Reference	Liu et al. (2022)
Barrier integrity and inflammatory tuning	
EV source	*Lactobacillus kefirgranum* PRCC-1301
Model	Caco-2 cells/DSS-related inflammatory models
Epithelial interface	Intestinal epithelium
Main reported effect	Attenuation of inflammatory responses and preservation of barrier-related markers
Reference	Kang et al. (2020)
EV source	*Lactobacillus paracasei*
Model	HT29 cells exposed to LPS
Epithelial interface	Intestinal epithelium
Main reported effect	Reduction of inflammatory mediators
Reference	Choi et al. (2020)
EV source	*Lactobacillus rhamnosus* GG
Model	DSS-induced colitis mouse model
Epithelial interface	Colon/intestinal barrier
Main reported effect	Attenuation of colitis-associated inflammation
Reference	Tong et al. (2021)
EV source	*Lactobacillus plantarum* Q7
Model	DSS-induced colitis mouse model
Epithelial interface	Colon/intestinal barrier
Main reported effect	Improvement of colitis symptoms and reduced cytokine production
Reference	Hao et al. (2021)
Epithelial repair and remodeling	
EV source	*Lactobacillus casei* BL23
Model	Intestinal epithelial model
Epithelial interface	Intestinal barrier/repair
Main reported effect	Stimulation of epithelial repair-associated signaling
Reference	Bäuerl et al. (2020)
EV source	*Lactobacillus rhamnosus* GG
Model	Full-thickness wound-healing mouse model
Epithelial interface	Skin repair/re-epithelialization
Main reported effect	Promotion of wound repair
Reference	Wang et al. (2024)
Adhesion, colonization resistance and pathogen exclusion	
EV source	*Lactobacillus crispatus* BC5/*Lactobacillus gasseri* BC12
Model	Cervicovaginal epithelial cells/HeLa adhesion model
Epithelial interface	Vaginal/urogenital epithelium
Main reported effect	Enhanced adhesion of beneficial lactobacilli and inhibition of pathogen attachment
Reference	Croatti et al. (2022)
EV source	Vaginal lactobacilli
Model	Human tissues and isolated cells
Epithelial interface	Vaginal mucosa
Main reported effect	Inhibition of HIV-1 infection
Reference	Ñahui Palomino et al. (2019)
Epithelial protection and epithelial-immune dialogue	
EV source	*Lactobacillus plantarum*
Model	HaCaT cells, macrophages and atopic dermatitis mouse model
Epithelial interface	Skin barrier
Main reported effect	Protection against *Staphylococcus aureus* EV-induced inflammatory damage
Reference	Kim et al. (2018)
EV source	*Lactobacillus plantarum*
Model	THP-1/skin-associated immune model
Epithelial interface	Skin immune interface
Main reported effect	Promotion of anti-inflammatory M2-like polarization
Reference	Kim et al. (2020) and Gong et al. (2024)

## 4 Prospective A549 framework: early epithelial transcriptional remodeling

While LAB-derived EV activity has been mainly investigated in intestinal, vaginal and skin-related epithelial models, their impact on the respiratory epithelium remains comparatively less defined. This gap is relevant because respiratory epithelial cells represent an additional mucosal interface in which barrier organization, cell–matrix adhesion, microbial sensing and repair-associated pathways are tightly integrated. In this context, A549 cells were used as an exploratory respiratory epithelial model to evaluate whether LAB-derived EVs may modulate transcriptional programs associated with epithelial interface homeostasis, as detailed in the Supplementary methods.

A549 cells were selected as a first-line exploratory respiratory epithelial model because they provide a robust, reproducible and widely used alveolar epithelial-like system for studying early epithelial responses, respiratory stress signaling and host–microbe interactions (Foster et al., 1998). In the context of this Perspective, A549 cells were not used to demonstrate definitive barrier reinforcement, but to explore whether LAB-derived EV-enriched preparations could induce early transcriptional responses related to epithelial interface homeostasis. The limitations of this model should be explicitly acknowledged. A549 cells are adenocarcinoma-derived and are generally cultured under submerged conditions. They do not fully reproduce the differentiation state, polarity or barrier properties of primary alveolar or airway epithelial cells, and they display limited transepithelial barrier function compared with primary respiratory epithelial cultures or air–liquid interface models. Therefore, the present A549 dataset should be interpreted as an exploratory transcriptional screening model and not as a functional demonstration of improved respiratory epithelial barrier activity.

*Lactobacillus fermentum* 18A-TV was selected because it was available within our collaborative framework, generated a detectable EV-enriched preparation under the tested culture conditions and represents a biologically relevant LAB strain for epithelial interaction studies. The strain has been described in relation to adhesive and pathogen-exclusion properties, supporting its suitability for an exploratory epithelial-interface model. However, EVs specifically derived from *L. fermentum* 18A-TV have not yet been extensively characterized in the literature. Accordingly, no strain-specific EV mechanism is claimed here. The use of this strain should therefore be interpreted as an exploratory starting point, requiring future validation through orthogonal EV characterization, more purified or fractionated preparations, dose–response studies and functional epithelial assays.

A549 cells were exposed to an EV-enriched preparation obtained from *L. fermentum* 18A-TV after 60 h of culture. This condition was selected because, among the tested culture time points, the 60 h preparation showed the strongest vesicle-associated signal for this strain. Briefly, *L. fermentum* 18A-TV was grown in MRS broth at 37 °C and the 60 h culture was processed by centrifugation and filtration to obtain a cell-free supernatant, followed by sequential ultracentrifugation to recover an EV-enriched pellet. The 60 h culture showed OD_600_ 2.234, pH 4.30 and 4 × 10^^8^ CFU/mL. The corresponding EV-enriched preparation showed a derived count rate of 797.4, a predominant nanoscale population by number distribution around 46.8 nm, protein content of 1,424 ng/μL and DNA content of 0.804 ng/μL. These parameters supported the selection of this fraction for exploratory epithelial assays, while requiring cautious interpretation because additional orthogonal characterization and purification controls are needed before assigning the observed effects specifically to vesicular components.

The transcriptional response was analyzed by RT-qPCR at 1 h, 3 h and 24 h. The gene panel was designed to explore complementary components of the epithelial interface, including tight-junction and junctional molecules, adherens-junction markers, extracellular matrix components, integrin subunits and selected growth/remodeling mediators. This design was intended to capture the temporal organization of the epithelial response rather than a single endpoint. For data handling, each experimental set, labeled A–G, was considered independently. Values were interpreted as matched EVs-versus-dPBS changes within the corresponding experimental set and time point. For graphical and biological readability, ΔΔCt values were converted to log_2_ fold-change according to the relationship log_2_FC = −ΔΔCt. Positive log_2_FC values therefore indicate higher transcript abundance in EV-treated cells compared with matched dPBS controls, whereas negative values indicate lower transcript abundance.

**Figure 1:**
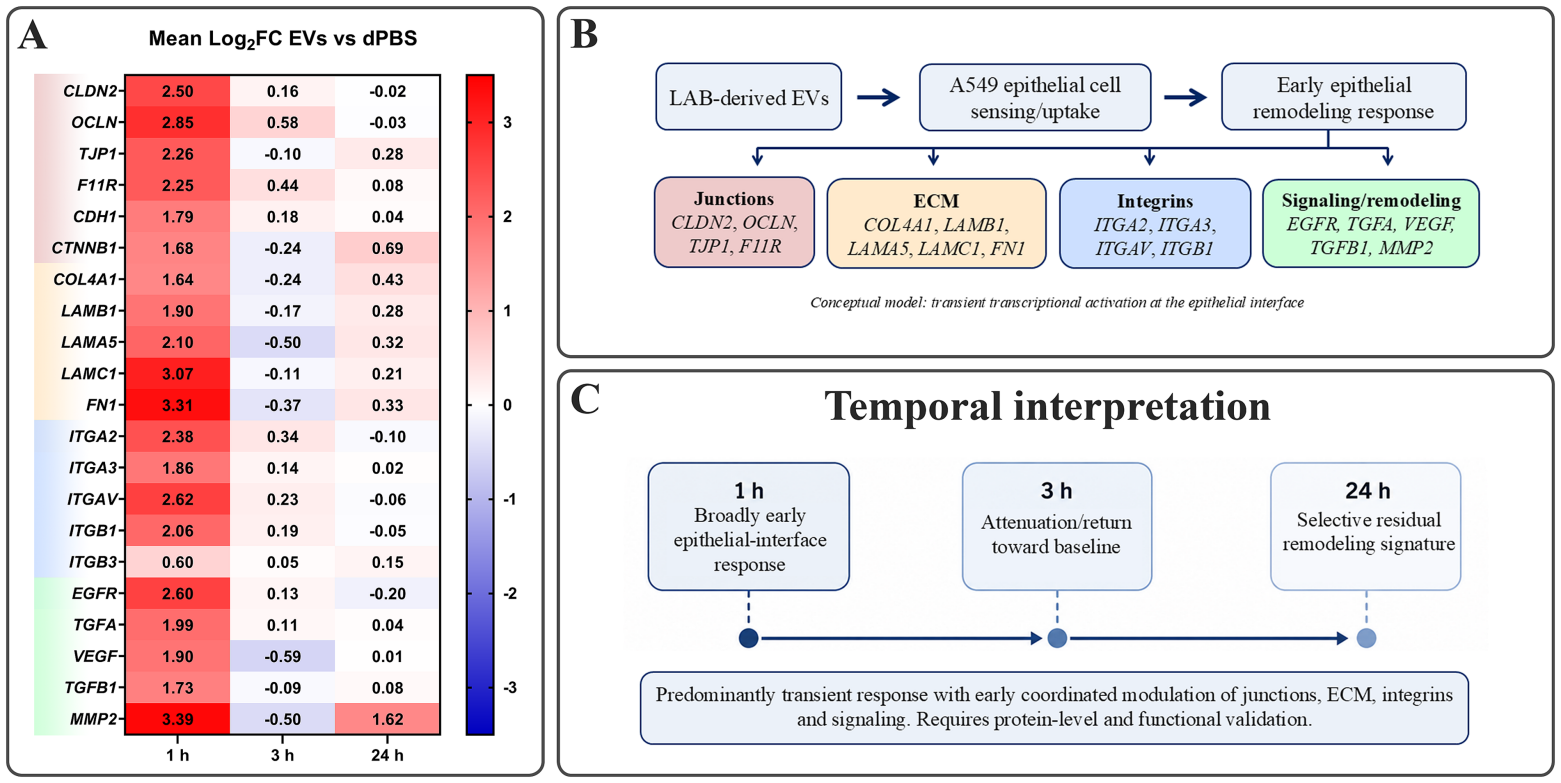
Prospective model of LAB-derived EV-induced epithelial interface remodeling in A549 cells. **(A)** Mean log_2_ fold-change (FC) heatmap summarizing the exploratory RT-qPCR response of A549 cells exposed to *Lactobacillus fermentum* 18A-TV-derived EV-enriched preparations at 1 h, 3 h and 24 h. Positive log_2_FC values indicate higher transcript abundance in EV-treated cells compared with matched dPBS controls, whereas negative values indicate lower transcript abundance. **(B)** Conceptual model showing the proposed interaction between LAB-derived EVs and the epithelial interface. LAB-derived EVs are proposed to act through epithelial sensing and/or uptake, leading to early modulation of complementary epithelial-interface modules, including junctional organization, extracellular matrix-associated genes, integrin-related transcripts/cell–matrix adhesion-associated genes and growth/remodeling-associated signaling. **(C)** Schematic interpretation of the temporal response, highlighting a broad early epithelial-interface signature at 1 h, attenuation toward baseline at 3 h and a more selective residual remodeling signature at 24 h. The model is hypothesis-generating and requires confirmation by protein-level and functional assays.

The most coherent signal was observed at 1 h. At this early time point, the response was not restricted to a single gene class, but involved a broader epithelial-interface signature. Junctional and barrier-associated genes, including *CLDN2*, *OCLN*, *TJP1* and *F11R*, showed positive log_2_FC values, consistent with early modulation of cell–cell junctional organization. In parallel, extracellular matrix-associated genes, including *COL4A1*, *LAMB1*, *LAMA5*, *LAMC1* and *FN1*, together with integrin subunits such as *ITGA2*, *ITGA3*, *ITGAV* and *ITGB1*, were consistent with an early integrin-related and matrix-associated transcriptional response. Crucially, these transcriptomic variations must be considered strictly hypothesis-generating, as they represent early regulatory changes that do not automatically imply or predict definitive changes in protein abundance, structural assembly, or actual functional outcomes at the epithelial barrier. This observation should be interpreted as a hypothesis-generating signal and not as evidence of altered integrin protein abundance, integrin activation or functional cell–matrix adhesion. Growth and remodeling-associated mediators, including *EGFR*, *TGFA*, *VEGF*, *TGFB1* and *MMP2*, were also included in the early remodeling-associated transcriptional signature (Figures 1A,B).

At 3 h, most genes returned toward baseline or displayed low-magnitude changes, suggesting that the response was largely transient (Figure 1C). At 24 h, the transcriptional profile appeared more selective, with residual modulation of selected targets such as *MMP2*, *CTNNB1*, *F11R*, *COL4A1* and *OCLN* in some experimental sets. Among remodeling-associated genes, *MMP2* showed modulation within the early transcriptional response. This finding should be interpreted cautiously as an indication of possible extracellular matrix turnover-related signaling, and not as evidence of increased *MMP2* protein abundance, enzymatic activity or matrix degradation. Overall, this temporal profile supports a prospective model in which *L. fermentum* 18A-TV EV-enriched preparations are associated with a rapid epithelial transcriptional signature, compatible with epithelial sensing and remodeling-associated responses, that attenuates within a few hours and is followed by a more restricted late transcriptional signature. These observations should be interpreted conservatively. Not all genes were analyzed in all experimental sets, and some targets are represented by single observations. Therefore, the dataset is best used to identify directionality, magnitude and temporal coherence of the response, rather than to support formal statistical inference. In addition, *ACTB* and *GAPDH* displayed early modulation in some experimental sets and were therefore not considered biological endpoints in the main interpretation. Accordingly, the present ΔΔCt-based analysis should be considered provisional until stable reference genes, or the geometric mean of multiple validated reference genes, are confirmed, in line with general RT-qPCR reporting and normalization principles (Vandesompele et al., 2002; Bustin et al., 2009). Thus, the A549 data should be viewed as hypothesis-generating evidence supporting the need for protein-level and functional validation of LAB-derived EV-induced epithelial remodeling.

## 5 Future directions and standardization needs

The evidence discussed in this Perspective supports the view that LAB-derived EVs should be interpreted as context-dependent postbiotic particles acting at the epithelial interface. Their effects are unlikely to correspond to a single conserved molecular output across all tissues, strains and preparations. Instead, both the available literature and the present exploratory A549 observations suggest a recurrent functional target: the regulation of epithelial interface homeostasis, including junctional organization, cell–matrix adhesion, epithelial–immune communication and tissue repair. In epithelial systems, barrier function depends on the integration of tight junctions, adherens junctions, cytoskeletal organization, extracellular matrix anchoring and local signaling cues. For this reason, a barrier-centered interpretation of LAB-EV activity should not be restricted to classical tight-junction markers alone. The early A549 response is compatible with this broader view because genes associated with junctions, extracellular matrix components, integrins and repair-associated signaling were modulated in a temporally coordinated manner. This pattern suggests that LAB-derived EVs may transiently sensitize epithelial cells to a remodeling state rather than simply increasing or decreasing isolated barrier-related transcripts. At the same time, the present observations remain exploratory. The A549 dataset does not demonstrate improved barrier function, increased protein expression or measurable epithelial protection. Rather, it identifies a transcriptional signature compatible with early epithelial remodeling. The absolute lack of protein-level confirmation (e.g., via Western blot or immunofluorescence) and the absence of key functional barrier assays, such as Transepithelial Electrical Resistance (TEER) measurements or paracellular tracer permeability assays, represent a major limitation of this preliminary dataset. The next step is therefore to connect this signature to functional phenotypes, including tight-junction localization, epithelial permeability, integrin expression, cell–matrix adhesion, migration, wound-repair capacity and viability or cytotoxicity endpoints. In particular, the integrin-related component of the A549 response should be validated by measuring integrin protein expression and localization, focal adhesion-associated signaling, cell–matrix adhesion and migration, ideally including perturbation approaches such as integrin-blocking antibodies or matrix-specific adhesion assays. A second priority is methodological standardization. Because bacterial EV preparations may contain co-isolated soluble microbial products, protein aggregates or medium-derived particles, future studies should include EV-depleted controls, medium-only particle controls, orthogonal EV characterization and dose–response designs. These controls are essential to distinguish true EV-mediated effects from responses induced by contaminants or non-vesicular bacterial products. Furthermore, moving from simple ultrafiltration or ultracentrifugation toward high-resolution purification workflows, such as size-exclusion chromatography combined with density gradients, will be vital to ensure high batch-to-batch reproducibility and minimize confounding factors. In parallel, the use of stable reference genes should be validated before final RT-qPCR interpretation, particularly when common housekeeping genes show experimental modulation. Finally, variability among preparations and experimental sets should be treated as biologically informative rather than as an obstacle to interpretation. Importantly, because the adenocarcinoma-derived A549 cell line lacks the complex cell differentiation and physiological architecture of the native lung tissue, future validation must transition to more complex and translationally relevant models. Validating these exploratory findings in primary human respiratory epithelial cells cultured at the air–liquid interface (ALI) or in specialized lung organoid systems will be essential to establish the true physiological relevance of LAB-derived EVs. Future work should define which components of the response are reproducible across batches and which depend on vesicle preparation, bacterial strain, dose or epithelial state. This will be particularly important for translational development, where reproducibility, potency assays and standardized manufacturing will be required before LAB-derived EVs can be considered reliable postbiotic candidates.

## 6 Concluding remarks

LAB-derived EVs are emerging as cell-free microbial effectors with potential postbiotic relevance at epithelial surfaces. The currently available evidence, particularly from intestinal, vaginal and skin-related models, indicates that these vesicles may influence barrier-associated pathways, inflammatory tone, pathogen exclusion and tissue repair. Within this framework, the present exploratory A549 observations suggest that alveolar epithelial-like cells may also mount a rapid and transient transcriptional response involving junctional components, extracellular matrix-associated genes, integrin-related transcripts and remodeling-associated mediators.

Taken together, these observations support a perspective in which LAB-derived EVs should not be regarded as passive microbial debris or as uniformly beneficial anti-inflammatory particles. Rather, they may act as context-dependent biological signals whose recurrent functional target is the epithelial interface. Their effects are likely to vary according to bacterial strain, vesicle cargo, preparation method and target tissue, rendering any overgeneralized assumption scientifically inaccurate. Every LAB strain possesses a distinct vesicular fingerprint, but the most consistent functional domains emerging across studies include barrier regulation, adhesion, epithelial–immune dialogue and repair-associated responses.

This conceptual framework provides a rationale for future mechanistic and functional studies aimed at defining how LAB-derived EVs regulate epithelial homeostasis and whether such effects can be translated into robust postbiotic applications.

## Data Availability

The raw data supporting the conclusions of this article will be made available by the authors, without undue reservation. Requests to access these datasets should be directed to the corresponding author.
