# Supplementary material for: Lactic acid bacteria-derived extracellular vesicles as postbiotic modulators of epithelial adhesion and barrier homeostasis: emerging perspectives
Source: Front Microbiol. 2026 Jul 29;17:1905504. doi: 10.3389/fmicb.2026.1905504 (PMC13461707; doi:10.3389/fmicb.2026.1905504)
Supplement: Supplementary file 1 [file Data_Sheet_1.DOCX]

Supplementary Material

**Supplementary Methods**

**Preparation of the *Lactobacillus fermentum* 18A-TV EV-enriched fraction**

*Lactobacillus fermentum* 18A-TV was grown in the Man, Rogosa, and Sharpe (MRS) medium broth at 37°C. For the exploratory epithelial assays described in this Perspective, the EVs-enriched preparation obtained from the 60 h culture was selected because this condition showed the strongest vesicle-associated signal among the tested culture time points. The 60 h culture was processed by centrifugation at 3,220 × g for 1h at 4°C to remove bacterial cells, followed by filtration through 0.22 µm filters to obtain a cell-free supernatant. EV-enriched pellets were then recovered by sequential ultracentrifugation at 100,000 × g for 60 min and 134,700 × g for 40 min at 4°C. Pellets were resuspended in sterile PBS and stored at −80°C until use.

The 60 h *L. fermentum* 18A-TV culture showed OD_600_ 2.234, pH 4.30 and 4 × 10^8^ CFU/mL. The corresponding EVs-enriched preparation showed a derived count rate of 797.4, a predominant nanoscale population by number distribution around 46.8 nm, protein content of 1424 ng/µL and DNA content of 0.804 ng/µL. These parameters support the use of the preparation as a EV-enriched fraction for exploratory assays, while recognizing that additional orthogonal characterization and purification controls are required before assigning the observed biological effects exclusively to purified vesicles.

**A549 epithelial cell exposure**

A549 human alveolar epithelial cells were used as an exploratory respiratory epithelial model. Cells were seeded at 5 x 10^5^ cells in 12-well plates and maintained in Roswell Park Memorial Institute (RPMI) with 10% Fetal Bovine Serum (FBS) under standard culture conditions. For stimulation experiments, cells were exposed to the *L. fermentum* 18A-TV EV-enriched preparation or matched dPBS control for 1 h, 3 h and 24 h. The EVs preparation was used at 5,44 x 10^12^ EVs per well for each time point, cells were collected for RNA extraction.

**RNA extraction, cDNA synthesis and RT-qPCR**

The RT-qPCR panel was designed to explore complementary components of the epithelial interface, including junctional/barrier genes, extracellular matrix-associated genes, integrin subunits and selected growth/remodeling mediators. The functional organization of the gene panel is reported in **Supplementary Table S1**. Cell samples were homogenized using QIAshredder (Qiagen) and total RNA was purified using RNeasy Kit (Qiagen), according to the manufacturer’s instructions. RNA quantity and quality were assessed by Qubit™ RNA BR Assay Kits (Invitrogen) and agarose gel electrophoresis, respectively. cDNA was synthesized from 500 ng of total RNA using M-MLV Reverse Transcriptase (Invitrogen). RT-qPCR was performed using SsoAdvanced Universal SYBR Green Supermix (BioRad) with primers listed in **Supplementary Table S2**.

**Data handling and exploratory interpretation**

Each letter from A to G identifies an independent experimental set. Values were interpreted as matched EVs-versus-dPBS changes within the corresponding experimental set and time point. For graphical and biological readability, ΔΔCt values were converted to log_2_ fold-change according to the relationship: log_2_FC = −ΔΔCt.

Positive log_2_FC values indicate higher transcript abundance in EV-treated cells compared with matched dPBS controls, whereas negative values indicate lower transcript abundance.

Because not all genes were analyzed in all experimental sets and some targets are represented by single observations, the dataset was used to identify directionality, magnitude and temporal coherence of the response rather than to support formal statistical inference. *ACTB* and *GAPDH* were retained in the complete dataset for transparency, but they were not considered biological endpoints in the main interpretation because they displayed early modulation in some experimental sets. Before definitive normalization and quantitative conclusions are drawn, reference gene stability should be validated using appropriate algorithms or multiple reference genes.

The complete ΔΔCt and log_2_FC dataset is provided in **Supplementary Table S3**. Dot plots showing individual experimental sets and mean log_2_FC values at 1 h, 3 h and 24 h are provided in **Supplementary Figure S1**.

**Supplementary Captions**

**Supplementary Table S1.** Functional organization of the A549 RT-qPCR gene panel used to explore LAB-EVs-induced epithelial responses.

**Supplementary Table S2.** RT-qPCR primer sequences used to analyze A549 epithelial responses.

**Supplementary Table S3.** Complete A549 RT-qPCR dataset used for exploratory interpretation. Values are reported as matched ΔΔCt (EVs minus dPBS) and as log_2_FC EVs vs dPBS, calculated as log_2_FC = −ΔΔCt. *ACTB* and *GAPDH* are included for transparency but should not be used as biological endpoints in the main interpretation.

**Supplementary Figure S1. Dot plots of the A549 RT-qPCR response to LAB-derived EVs at 1 h, 3 h and 24 h.** Each color represents one independent experimental set (Exp A – Exp G). The violin plot bar indicates the mean log_2_FC for genes, while each colored dot represents the exact value for each experimental set at the indicated time point. Positive values indicate increased transcript abundance in EVs-treated cells compared with matched dPBS controls, whereas negative values indicate lower transcript abundance. *ACTB* and *GAPDH* are excluded because their stability as reference genes requires further assessment.
