# Supplementary material for: Lactic acid bacteria-derived extracellular vesicles as postbiotic modulators of epithelial adhesion and barrier homeostasis: emerging perspectives
Source: Front Microbiol. 2026 Jul 29;17:1905504. doi: 10.3389/fmicb.2026.1905504 (PMC13461707; doi:10.3389/fmicb.2026.1905504)
Supplement: Supplementary file 2 [file Table_1.DOCX]

Supplementary Material

**Supplementary Table S1**

| **Functional area** | **Genes** | **Biological meaning** |
| --- | --- | --- |
| Junctional / barrier organization | *CLDN2*, *OCLN*, *TJP1*, *F11R*, *CDH1*, *CTNNB1* | Cell-cell cohesion, tight and adherens junction organization, epithelial polarity and barrier-associated interface regulation |
| Extracellular matrix | *COL4A1*, *LAMB1*, *LAMA5*, *LAMC1*, *FN1* | Basement membrane components, matrix anchoring and epithelial structural remodeling |
| Integrins / cell-matrix adhesion | *ITGA1*, *ITGA2*, *ITGA3*, *ITGAV*, *ITGB1*, *ITGB3* | Cell-matrix adhesion, mechanosensing, survival signaling and repair-associated epithelial responses |
| Growth / remodeling signaling | *EGFR*, *TGFA*, *VEGF*, *TGFB1*, *MMP2* | Repair-associated signaling, adaptive remodeling, matrix turnover and epithelial stress-response pathways |
| Reference / reporter genes | *ACTB*, *GAPDH* | Candidate reference genes included for transparency; their stability requires validation before use for definitive normalization |
