# Supplementary material for: Lactic acid bacteria-derived extracellular vesicles as postbiotic modulators of epithelial adhesion and barrier homeostasis: emerging perspectives
Source: Front Microbiol. 2026 Jul 29;17:1905504. doi: 10.3389/fmicb.2026.1905504 (PMC13461707; doi:10.3389/fmicb.2026.1905504)
Supplement: Supplementary file 3 [file Table_2.DOCX]

Supplementary Material

**Supplementary Table S2**

| **Gene** | **Functional group** | **Forward primer (5'-3')** | **Reverse primer (5'-3')** | **Amplicon size (bp)** | **GeneID** |
| --- | --- | --- | --- | --- | --- |
| *CLDN2* | Junctional / barrier organization | AGTCTTCTGCCAGGAATCC | CGTAGGATCCCATGAAGATTCC | 120 | 9075 |
| *OCLN* | Junctional / barrier organization | AGACCCAAGAGCAGCAAAGG | ACCATGATGCCCAGGATAGC | 162 | 100506658 |
| *TJP1* | Junctional / barrier organization | CAATGGAGGAAACAGCTATATGG | ATCTCGTCCACCAGATATTGC | 95 | 7082 |
| *F11R* | Junctional / barrier organization | GATAGTGATGCCTACGAATCC | GTGTCCCATACCCATTCC | 152 | 50848 |
| *CDH1* | Junctional / barrier organization | CGTACACAGCCCTAATCATAGC | ATAGTTCGAGGTTCTGGTATGG | 121 | 999 |
| *CTNNB1* | Junctional / barrier organization | CAAGCCACAAGATTACAAGAAACG | CCAAGATCAGCAGTCTCATTCC | 96 | 1499 |
| *COL4A1* | Extracellular matrix | GGAGACCCTGGCTTGAAAGG | CTGCCCATGTCCACCTTATCC | 83 | 1282 |
| *LAMB1* | Extracellular matrix | CCAACAGGACACACAGATTCC | TCCACAGTCTCACGGTAAGG | 82 | 3912 |
| *LAMA5* | Extracellular matrix | CGCCTTCTACAGCAATGC | AGCCACTGAAGTTGTAAATGG | 170 | 3911 |
| *LAMC1* | Extracellular matrix | AGAAGGCTTTGTGGGAAATCG | TGATCAGCAACCTTATCCTTTACC | 123 | 3915 |
| *FN1* | Extracellular matrix | TAAGCTGTACCATCGCAAACC | ACACACACACTCTAACATGTAACC | 110 | 2335 |
| *ITGA2* | Integrins / cell-matrix adhesion | AAGTGAACAGTTTGGCTATGC | GACATTTATACACATCTCCCATTCG | 119 | 3673 |
| *ITGA3* | Integrins / cell-matrix adhesion | CAAGTGGCTGCTGTATCC | AGGTTGAGAGGGTTGATAAGG | 93 | 3675 |
| *ITGAV* | Integrins / cell-matrix adhesion | GGCAAGGTCAGCTTATTTCG | CAGTCCGAGTTGCTAATTGG | 102 | 3685 |
| *ITGB1* | Integrins / cell-matrix adhesion | TTCAAGAGAGCTGAAGACTATCC | CAATTCTGAAGTCCGAAGTAATCC | 143 | 3688 |
| *ITGB3* | Integrins / cell-matrix adhesion | GCTGATGACTGAGAAGCTATCC | GGAGGACATTGCTGGAATCC | 146 | 3690 |
| *EGFR* | Growth / remodeling signaling | ATAGAAATGGGCTGCAAAGC | AGGAAGGTGTCGTCTATGC | 109 | 1956 |
| *TGFA* | Growth / remodeling signaling | GGTCCGAAAACACTGTGAGTGG | CAAACTCCTCCTCTGGGCTCTT | 130 | 7039 |
| *VEGF* | Growth / remodeling signaling | GGGAGCTTCAGGACATTGC | CAGAAGCAGGTGAGAGTAAGC | 128 | 7422 |
| *TGFB1* | Growth / remodeling signaling | ACTCATTCAGTCACCATAGC | CTCTCTGGGCTTGTTTCC | 85 | 7040 |
| *MMP2* | Growth / remodeling signaling | GAGCATGGCGATGGATACC | CTTCTCCCAAGGTCCATAGC | 124 | 4313 |
| *ACTB* | Reference / reporter gene | CACCATTGGCAATGAGCGGTTC | AGGTCTTTGCGGATGTCCACGT | 135 | 60 |
| *GAPDH* | Reference / reporter gene | TCATCCATGACAACTTTGGTATCG | CCATCACGCCACAGTTTCC | 109 | 2597 |
