# Supplementary material for: Lactic acid bacteria-derived extracellular vesicles as postbiotic modulators of epithelial adhesion and barrier homeostasis: emerging perspectives
Source: Front Microbiol. 2026 Jul 29;17:1905504. doi: 10.3389/fmicb.2026.1905504 (PMC13461707; doi:10.3389/fmicb.2026.1905504)
Supplement: Supplementary file 4 [file Table_3.DOCX]

Supplementary Material

**Supplementary Table S3**

| **Gene** | **Functional group** | **Time** | **Exp.** | **ΔΔCt**  **EVs-dPBS** | **log2FC**  **EVs vs dPBS** |
| --- | --- | --- | --- | --- | --- |
| *CLDN2* | Junctional/barrier | 1 h | B | -1.85 | +1.85 |
| *CLDN2* | Junctional/barrier | 1 h | E | -3.15 | +3.15 |
| *CLDN2* | Junctional/barrier | 3 h | B | -0.15 | +0.15 |
| *CLDN2* | Junctional/barrier | 3 h | E | -0.17 | +0.17 |
| *CLDN2* | Junctional/barrier | 24 h | B | +0.44 | -0.44 |
| *CLDN2* | Junctional/barrier | 24 h | E | -0.40 | +0.40 |
| *OCLN* | Junctional/barrier | 1 h | B | -2.40 | +2.40 |
| *OCLN* | Junctional/barrier | 1 h | E | -3.82 | +3.82 |
| *OCLN* | Junctional/barrier | 1 h | G | -2.33 | +2.33 |
| *OCLN* | Junctional/barrier | 3 h | B | -0.57 | +0.57 |
| *OCLN* | Junctional/barrier | 3 h | E | -0.96 | +0.96 |
| *OCLN* | Junctional/barrier | 3 h | G | -0.22 | +0.22 |
| *OCLN* | Junctional/barrier | 24 h | B | +0.22 | -0.22 |
| *OCLN* | Junctional/barrier | 24 h | E | -0.51 | +0.51 |
| *OCLN* | Junctional/barrier | 24 h | G | +0.38 | -0.38 |
| *TJP1* | Junctional/barrier | 1 h | C | -2.26 | +2.26 |
| *TJP1* | Junctional/barrier | 3 h | C | +0.10 | -0.10 |
| *TJP1* | Junctional/barrier | 24 h | C | -0.33 | +0.33 |
| *TJP1* | Junctional/barrier | 24 h | D | -0.24 | +0.24 |
| *F11R* | Junctional/barrier | 1 h | B | -2.23 | +2.23 |
| *F11R* | Junctional/barrier | 1 h | G | -2.27 | +2.27 |
| *F11R* | Junctional/barrier | 3 h | B | -0.46 | +0.46 |
| *F11R* | Junctional/barrier | 3 h | G | -0.41 | +0.41 |
| *F11R* | Junctional/barrier | 24 h | B | +0.36 | -0.36 |
| *F11R* | Junctional/barrier | 24 h | D | -0.64 | +0.64 |
| *F11R* | Junctional/barrier | 24 h | G | +0.04 | -0.04 |
| *CDH1* | Junctional/barrier | 1 h | B | -1.79 | +1.79 |
| *CDH1* | Junctional/barrier | 3 h | B | -0.18 | +0.18 |
| *CDH1* | Junctional/barrier | 24 h | B | +0.26 | -0.26 |
| *CDH1* | Junctional/barrier | 24 h | D | -0.34 | +0.34 |
| *CTNNB1* | Junctional/barrier | 1 h | C | -1.68 | +1.68 |
| *CTNNB1* | Junctional/barrier | 3 h | C | +0.24 | -0.24 |
| *CTNNB1* | Junctional/barrier | 24 h | C | -0.58 | +0.58 |
| *CTNNB1* | Junctional/barrier | 24 h | D | -0.79 | +0.79 |
| *COL4A1* | Extracellular matrix | 1 h | C | -1.64 | +1.64 |
| *COL4A1* | Extracellular matrix | 3 h | C | +0.24 | -0.24 |
| *COL4A1* | Extracellular matrix | 24 h | C | -0.19 | +0.19 |
| *COL4A1* | Extracellular matrix | 24 h | D | -0.66 | +0.66 |
| *LAMB1* | Extracellular matrix | 1 h | C | -1.90 | +1.90 |
| *LAMB1* | Extracellular matrix | 3 h | C | +0.17 | -0.17 |
| *LAMB1* | Extracellular matrix | 24 h | C | -0.28 | +0.28 |
| *LAMA5* | Extracellular matrix | 1 h | C | -2.10 | +2.10 |
| *LAMA5* | Extracellular matrix | 3 h | C | +0.50 | -0.50 |
| *LAMA5* | Extracellular matrix | 24 h | C | -0.32 | +0.32 |
| *LAMC1* | Extracellular matrix | 1 h | C | -3.07 | +3.07 |
| *LAMC1* | Extracellular matrix | 3 h | C | +0.11 | -0.11 |
| *LAMC1* | Extracellular matrix | 24 h | C | -0.21 | +0.21 |
| *FN1* | Extracellular matrix | 1 h | C | -3.31 | +3.31 |
| *FN1* | Extracellular matrix | 3 h | C | +0.37 | -0.37 |
| *FN1* | Extracellular matrix | 24 h | C | -0.33 | +0.33 |
| *ITGA2* | Integrins/cell-matrix adhesion | 1 h | A | -2.28 | +2.28 |
| *ITGA2* | Integrins/cell-matrix adhesion | 1 h | F | -2.26 | +2.26 |
| *ITGA2* | Integrins/cell-matrix adhesion | 1 h | G | -2.58 | +2.58 |
| *ITGA2* | Integrins/cell-matrix adhesion | 3 h | A | +0.04 | -0.04 |
| *ITGA2* | Integrins/cell-matrix adhesion | 3 h | F | -0.14 | +0.14 |
| *ITGA2* | Integrins/cell-matrix adhesion | 3 h | G | -0.93 | +0.93 |
| *ITGA2* | Integrins/cell-matrix adhesion | 24 h | A | +0.61 | -0.61 |
| *ITGA2* | Integrins/cell-matrix adhesion | 24 h | F | -0.39 | +0.39 |
| *ITGA2* | Integrins/cell-matrix adhesion | 24 h | G | +0.07 | -0.07 |
| *ITGA3* | Integrins/cell-matrix adhesion | 1 h | A | -1.20 | +1.20 |
| *ITGA3* | Integrins/cell-matrix adhesion | 1 h | F | -2.52 | +2.52 |
| *ITGA3* | Integrins/cell-matrix adhesion | 3 h | A | -0.09 | +0.09 |
| *ITGA3* | Integrins/cell-matrix adhesion | 3 h | F | -0.18 | +0.18 |
| *ITGA3* | Integrins/cell-matrix adhesion | 24 h | A | +0.19 | -0.19 |
| *ITGA3* | Integrins/cell-matrix adhesion | 24 h | F | -0.22 | +0.22 |
| *ITGAV* | Integrins/cell-matrix adhesion | 1 h | A | -1.76 | +1.76 |
| *ITGAV* | Integrins/cell-matrix adhesion | 1 h | F | -4.09 | +4.09 |
| *ITGAV* | Integrins/cell-matrix adhesion | 1 h | G | -2.00 | +2.00 |
| *ITGAV* | Integrins/cell-matrix adhesion | 3 h | A | +0.01 | -0.01 |
| *ITGAV* | Integrins/cell-matrix adhesion | 3 h | F | -0.36 | +0.36 |
| *ITGAV* | Integrins/cell-matrix adhesion | 3 h | G | -0.34 | +0.34 |
| *ITGAV* | Integrins/cell-matrix adhesion | 24 h | A | +0.59 | -0.59 |
| *ITGAV* | Integrins/cell-matrix adhesion | 24 h | F | -0.52 | +0.52 |
| *ITGAV* | Integrins/cell-matrix adhesion | 24 h | G | +0.12 | -0.12 |
| *ITGB1* | Integrins/cell-matrix adhesion | 1 h | A | -1.90 | +1.90 |
| *ITGB1* | Integrins/cell-matrix adhesion | 1 h | B | -2.20 | +2.20 |
| *ITGB1* | Integrins/cell-matrix adhesion | 1 h | G | -2.09 | +2.09 |
| *ITGB1* | Integrins/cell-matrix adhesion | 3 h | A | -0.06 | +0.06 |
| *ITGB1* | Integrins/cell-matrix adhesion | 3 h | B | -0.40 | +0.40 |
| *ITGB1* | Integrins/cell-matrix adhesion | 3 h | F | 0.00 | 0.00 |
| *ITGB1* | Integrins/cell-matrix adhesion | 3 h | G | -0.31 | +0.31 |
| *ITGB1* | Integrins/cell-matrix adhesion | 24 h | A | +0.20 | -0.20 |
| *ITGB1* | Integrins/cell-matrix adhesion | 24 h | B | +0.32 | -0.32 |
| *ITGB1* | Integrins/cell-matrix adhesion | 24 h | F | -0.48 | +0.48 |
| *ITGB1* | Integrins/cell-matrix adhesion | 24 h | G | +0.15 | -0.15 |
| *ITGB3* | Integrins/cell-matrix adhesion | 1 h | A | -0.60 | +0.60 |
| *ITGB3* | Integrins/cell-matrix adhesion | 3 h | A | -0.05 | +0.05 |
| *ITGB3* | Integrins/cell-matrix adhesion | 24 h | A | +0.33 | -0.33 |
| *EGFR* | Growth/remodeling signaling | 1 h | A | -1.30 | +1.30 |
| *EGFR* | Growth/remodeling signaling | 1 h | B | -1.27 | +1.27 |
| *EGFR* | Growth/remodeling signaling | 1 h | C | -5.24 | +5.24 |
| *EGFR* | Growth/remodeling signaling | 3 h | A | -0.03 | +0.03 |
| *EGFR* | Growth/remodeling signaling | 3 h | B | -0.55 | +0.55 |
| *EGFR* | Growth/remodeling signaling | 3 h | C | +0.18 | -0.18 |
| *EGFR* | Growth/remodeling signaling | 24 h | A | +0.56 | -0.56 |
| *EGFR* | Growth/remodeling signaling | 24 h | B | +0.16 | -0.16 |
| *EGFR* | Growth/remodeling signaling | 24 h | C | -0.11 | +0.11 |
| *TGFA* | Growth/remodeling signaling | 1 h | A | -1.54 | +1.54 |
| *TGFA* | Growth/remodeling signaling | 1 h | C | -2.44 | +2.44 |
| *TGFA* | Growth/remodeling signaling | 3 h | A | -0.24 | +0.24 |
| *TGFA* | Growth/remodeling signaling | 3 h | C | +0.03 | -0.03 |
| *TGFA* | Growth/remodeling signaling | 24 h | A | +0.19 | -0.19 |
| *TGFA* | Growth/remodeling signaling | 24 h | C | -0.27 | +0.27 |
| *VEGF* | Growth/remodeling signaling | 1 h | A | -1.44 | +1.44 |
| *VEGF* | Growth/remodeling signaling | 1 h | C | -2.36 | +2.36 |
| *VEGF* | Growth/remodeling signaling | 3 h | A | -0.19 | +0.19 |
| *VEGF* | Growth/remodeling signaling | 3 h | C | +1.37 | -1.37 |
| *VEGF* | Growth/remodeling signaling | 24 h | A | +0.10 | -0.10 |
| *VEGF* | Growth/remodeling signaling | 24 h | C | -0.12 | +0.12 |
| *TGFB1* | Growth/remodeling signaling | 1 h | A | -1.66 | +1.66 |
| *TGFB1* | Growth/remodeling signaling | 1 h | C | -1.97 | +1.97 |
| *TGFB1* | Growth/remodeling signaling | 1 h | G | -1.56 | +1.56 |
| *TGFB1* | Growth/remodeling signaling | 3 h | A | +0.02 | -0.02 |
| *TGFB1* | Growth/remodeling signaling | 3 h | C | +0.49 | -0.49 |
| *TGFB1* | Growth/remodeling signaling | 3 h | G | -0.25 | +0.25 |
| *TGFB1* | Growth/remodeling signaling | 24 h | A | +0.18 | -0.18 |
| *TGFB1* | Growth/remodeling signaling | 24 h | C | -0.30 | +0.30 |
| *TGFB1* | Growth/remodeling signaling | 24 h | G | -0.12 | +0.12 |
| *MMP2* | Growth/remodeling signaling | 1 h | C | -3.39 | +3.39 |
| *MMP2* | Growth/remodeling signaling | 3 h | C | +0.50 | -0.50 |
| *MMP2* | Growth/remodeling signaling | 24 h | C | -1.62 | +1.62 |
